# *Tunicothrix halophila* n. sp., a Secondarily Oligomerized Parabirojimid Hypotrich (Ciliophora, Spirotrichea) From Hypersaline Costal Water in Korea

**DOI:** 10.3389/fmicb.2021.691361

**Published:** 2021-07-05

**Authors:** Atef Omar, Ji Hye Moon, Seung Won Nam, Jae-Ho Jung

**Affiliations:** ^1^Natural Science Research Institute, Gangneung-Wonju National University, Gangneung, South Korea; ^2^Department of Zoology, Al Azhar University, Assiut, Egypt; ^3^Department of Biology, Gangneung-Wonju National University, Gangneung, South Korea; ^4^Protist Research Team, Nakdonggang National Institute of Biological Resources, Sangju, South Korea

**Keywords:** ciliate, extreme habitat, ontogenesis, phylogeny, taxonomy

## Abstract

*Tunicothrix halophila* n. sp. was discovered in a hypersaline marine sample from Jeju Island, Korea. It is characterized by the highly reduced number of dorsal bristles. In addition, the main character of the genus *Tunicothrix* (e.g., alveolar layer) is absent/indistinct. To figure out its identity and phylogenetic relationship, we examined the new species based on modern morphological methods and molecular phylogenetic analyses. Since the parabirojimids are of basal position to core hypotrichs and a smaller data set could show incorrect phylogenetic relationships among the hypotrichs, we used a huge data set composed of 1,460 DNA sequences to infer the phylogenetic tree. The reduction of dorsal bristles is very likely a secondarily evolved character in hypotrichs, resulting in the independent phenotypic adaptation in the hypersaline ecosystems as shown in other hypersaline hypotrichs. Furthermore, the so-called right marginal row 1 in other congeners is found to produce a pretransverse and transverse cirrus and thus we recommend using the term frontoventral row. Based on our data, we can justify *Tunicothrix halophila* n. sp. as a new species; however, despite the phenotypic distinctiveness, we refrain to establish a new genus because of the missing data and the non-monophyly of *Tunicothrix*.

## Introduction

The hypersaline habitats harbor a high number of undescribed ciliates (Ruinen, 1938; Borror, 1972; Wilbert and Kahan, 1981, 1986; Wilbert, 1986, 1995; Foissner, 1987, 1999, 2012; Foissner et al., 2002; Berger, 2006, 2011; Li et al., 2019). However, only few hypotrichous genera have been discovered so far from such extreme environments, for instance *Afrothrix* Foissner, 1999; *Apourosomoida* Foissner et al., 2002; *Cladotricha* Gaievska a, 1925; *Erniella* Foissner, 1987; *Etoschothrix* Foissner et al., 2002; *Schmidingerothrix* Foissner, 2012; and *Schmidtiella* Li et al., 2019. In terms of morphology of the halophile hypotrichs, most of them have lost most or all dorsal bristles/kineties, which are rather equidistant and mostly bipolar in other hypotrich ciliates (Berger, 1999, 2006, 2011; Foissner, 2012). The reduced ciliary pattern of these taxa is suggested to be a secondary oligomerization that might be a common ancestor of hypotrichs, but it differs from the ancestor hypothesized by Berger (2008, 2011) and is very likely evolved independently among distinct taxa (Foissner et al., 2014; Li et al., 2019). This is supported by the discovery of *Schmidingerothrix extraordinaria* Foissner, 2012, an extraordinary hypotrich from African hypersaline soils, which lost some of the most stable features of hypotrichs such as all dorsal bristles, paroral, and the middle ciliary row of adoral membranelles. On the other hand, Li et al. (2019) discovered *Schmidtiella ultrahalophila* from a solar saltern. This species has few dorsal bristles and shows a basal position to other hypotrichs in the phylogenetic trees. However, the simplified character states and the fewer genetic data available in public database hamper researchers from inferring the common ancestor.

From a hypersaline marine sample, we discovered a new *Tunicothrix* ciliate with a highly reduced number of dorsal bristles that differs from other members of the family Parabirojimidae Dai and Xu, 2011, and is also very rare within hypotrichs (Berger, 1999, 2006, 2008, 2011; Dai and Xu, 2011; Foissner, 2012). Further, the cortical alveolar layer (*tunica*), which is one of the most prominent characters of the genus *Tunicothrix* Xu et al., 2006 (Lin and Song, 2004; Xu et al., 2006; Dai and Xu, 2011), is absent or significantly reduced and can be observed only at high magnification. Possibly, this is another adaptation to hypersaline habitat.

To figure out the identity and phylogenetic relationship of the new species, we examined the population based on modern morphological methods (live observations, protargol impregnation, and SEM) and molecular phylogenetic analyses. To examine the phylogenetic position of the new species, we used a huge data set composed of 1,460 DNA sequences because the parabirojimids are of basal positions to core hypotrichs and a smaller data set (commonly used in ciliate taxonomy/phylogeny) could show incorrect phylogenetic relationships among the hypotrichs. As we know, the hypotrichs are full of homoplasies/plesiomorphies and scattered in 18S rRNA gene trees. Based on our data, and despite the phylogenetic distinctiveness of *Tunicothrix halophila* n. sp., we refrain from establishing a new genus because of missing data and the non-monophyly of the genus *Tunicothrix*.

## Materials and Methods

### Sample Collection and Identification

*Tunicothrix halophila* n. sp. was discovered in a coastal water sample (34.9‰, 2.1°C at the time of sampling) from Jeju Island, Korea. The sample was collected in February 2020 and transferred to the laboratory within a week. It was maintained in a Petri dish (13.5 cm in diameter) for a year at room temperature (15–20°C). Sterilized wheat grains were supplied to enrich bacteria as a food source. After 10 months, the salinity became 71‰ due to evaporation. It should be noted that the new species was the only hypotrich found in the culture at that time.

The new species was examined under a stereomicroscope (SZ11; Olympus, Tokyo, Japan) and light microscopes (BX53, IX73; Olympus) using bright field and differential interference contrast (DIC) at magnifications of 50–1,000×. About 10 ml of the culture water was fixed using saturated HgCl_2_, and the cells were washed three to five times with tap water to remove the fixative by centrifugation (3,000–5,000 rpm, 30–60 s). The protargol impregnation was conducted using a synthesized protargol and acetone developer (Foissner, 2014; Kim and Jung, 2017). It should be noted that a new batch of the synthesized protargol powder showed an optimum impregnation condition at pH 8.5–8.7, which is distinctly higher than previous batches synthesized in our laboratory (e.g., below pH 8.0). Attempts to induce formation of resting cysts were unsuccessful. General terminology follows Lynn (2008), and specific terms of parabirojimids follow Dai and Xu (2011) except for the term “right marginal row 1” (for details, see “Discussion” section). For assigning/describing the buccal lips, we follow Foissner and Al-Rasheid (2006).

### DNA Extraction, PCR Amplification, and Sequencing

Five cells of *Tunicothrix halophila* n. sp. were isolated from the raw culture and washed with the culture water filtered by a 0.2-μm syringe filter (Minisart^®^ Syringe Filter; Sartorius, G ttingen, Germany) more than five times. Each cell was then transferred to a 1.5-ml tube using a micro-capillary with minimum volume of the water. Genomic DNA of each cell was extracted using a REDExtract-N-Amp Tissue PCR Kit (Sigma, St. Louis, MO, United States). The conditions for PCR were as follows: initial denaturation at 94°C for 1 min 30 s, followed by 40 cycles of denaturation at 98°C for 10 s, annealing at 58.5°C for 30 s, and extension at 72°C for 3 min, and a final extension step at 72°C for 7 min. The 18S rRNA gene was amplified using two slightly modified primers (New Euk A and LSU rev4) from Sonnenberg et al. (2007). After the amplification, the amplicons were purified using a MG PCR Purification Kit (MGmed, Seoul, Korea). We completed direct sequencing and assembled sequence fragments using one of the five cells because the five sequences determined by the New Euk A were completely identical. The DNA sequencing was performed using an ABI 3700 sequencer (Applied Biosystems, Foster City, CA, United States), and the following internal primers were used: 18SF790v2, 18SF1470, and 18SR300. Sequence fragments were assembled using Geneious Prime 2019.2.3 (Kearse et al., 2012).

### Scanning Electron Microscopy

Cells isolated from the raw culture were prepared for scanning electron microscopy following the protocol of Foissner (2014). Briefly, they were fixed for 30 min using an equal mix of 2% aqueous osmium tetroxide and 3% glutaraldehyde. The cells were attached on a coverslip using poly-L-lysine, dehydrated using an ethanol series (from 30% to 100%), and dried using a critical point dryer (EM CPD300, Leica, Vienna, Austria). Subsequently, they were coated with platinum using a sputter coater (EM SCD005, Leica) and JSM-IT500 (JEOL Ltd., Tokyo, Japan) was used for the observation.

### Phylogenetic Analyses

To infer the phylogenetic relationship of *Tunicothrix halophila* n. sp., we retrieved 1,459 18S rRNA gene sequences belonging to the class Spirotrichea from GenBank. As an outgroup, four species/sequences belonging to the class Protocruziea were selected. These sequences were aligned using ClustalW (Thompson et al., 1994) implemented into Geneious Prime 2019.2.3 (Kearse et al., 2012). Overhangs from both ends of the alignment were trimmed to construct blunt ends using Geneious, resulting in a final matrix of 1,955 columns. The best-fit substitution model for phylogenetic analysis, TVM + I (0.1260) + G (0.6620) based on the Akaike information criterion (AIC), was selected using jModelTest 2.1.10 (Guindon and Gascuel, 2003; Darriba et al., 2012). IQ-TREE 1.6.12 was used to infer the maximum likelihood (ML) trees, with 1,000 bootstrap replicates (Nguyen et al., 2015). The trees were visualized using FigTree 1.4.4^1^.

## Results

### ZooBank Registration

ZooBank registration number of present work: urn:lsid:zoobank.org:pub:7277C82F-18FF-483A-9747-BF22265B0518.

ZooBank registration number of *Tunicothrix halophila* n. sp.: urn:lsid:zoobank.org:act:D64923EA-0756-4347-9051-D1B1ADAF53C8.

### Taxonomy and Morphological Description of *Tunicothrix halophila* n. sp.

#### Diagnosis

Body size 45–70 × 12–18 μm *in vivo*, body outline elliptical to slightly slender with slightly narrow posterior end; cortex flexible, but not contractile. 2 macronuclear nodules. Arc-shaped structures underneath pellicle, 3.0–4.0 μm long *in vivo*. 3 frontal, 1 parabuccal, and 1 buccal cirrus on frontoventral area; midventral complex composed of 1 cirral pair and 1 cirral row with 3–5 cirri; 1 frontoventral row with 9–12 cirri; 1 pretransverse, 3 transverse cirri; 1 left and 1 right marginal row, left marginal row distinctly shortened posteriorly. 3 dorsal kineties with sparse cilia; caudal cirri lacking. Bipartite adoral zone of membranelles composed of invariably 4 frontal and 9 ventral membranelles; undulating membranelles slightly curved leftward and optically crossing.

#### Type Locality

Hypersaline water collected from a puddle on a harbor in Jeju Island, South Korea (33°31′9″N, 126°31′44″E). This temporary puddle is right next to the seaside (<1 m) and formed by the waves crashing into the harbor and/or the water from fishermen unloading fishery products. As mentioned in the material and methods section, the salinity was 34.9‰ at the sampling site and the new species was absent at the beginning of the culture. By the evaporation for months in the laboratory, the salinity became high and the species appeared. *Tunicothrix halophila* n. sp. is very likely a hypersaline species well adapted to temporary habitats in the coastal area because there is no stable hypersaline ecosystem (e.g., solar saltern) near the type locality.

#### Type Material

The slide containing the holotype (NNIBRPR17559) and three paratype slides (NNIBRPR17560–17562) with protargol-impregnated specimens have been deposited in the Nakdonggang National Institute of Biological Resources, South Korea. Another paratype slide (GUC004219) has been deposited in the Jung-lab (J.-H. Jung) in Gangneung-Wonju National University.

#### Etymology

The species-group name *halophila* (feminine) is a composite of the Greek words *halós* (salt) and *philos* (preferring), referring to the hypersaline habitat where the species occurs.

#### Description

**FIGURE 1:**
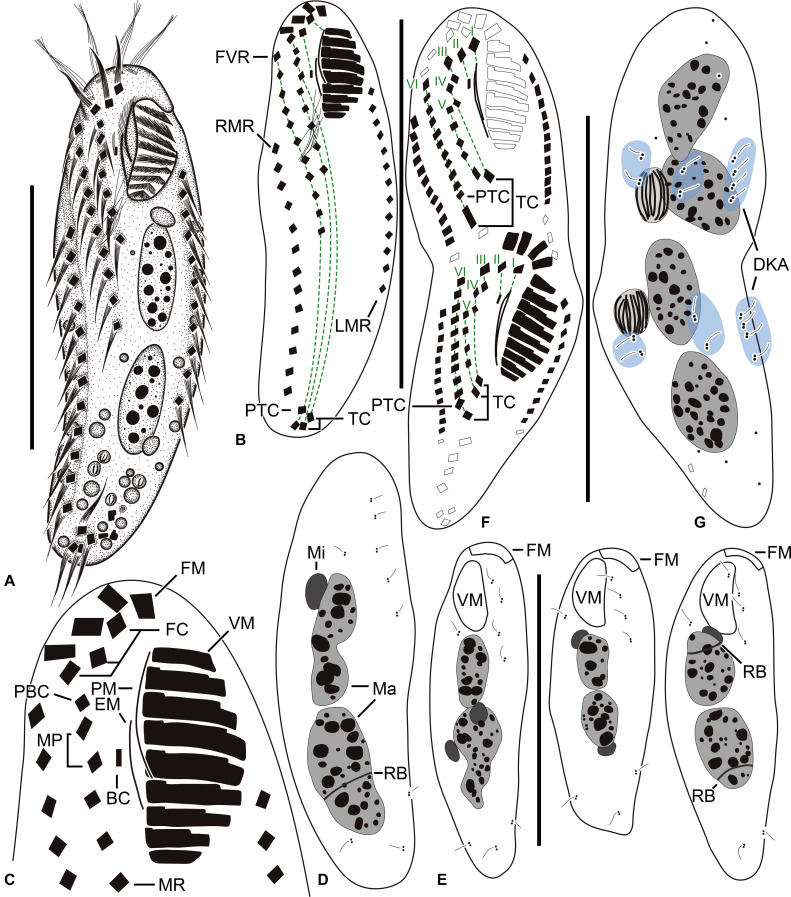
*Tunicothrix halophila* n. sp. *in vivo* **(A)** and after protargol impregnation **(B–G)**. **(A)** Ventral view of a representative specimen. **(B–D)** Ventral **(B,C)** and dorsal **(D)** views of the holotype specimen. **(E)** Dorsal views showing dorsal bristles and nuclear apparatus. **(F,G)** Ventral **(F),** and dorsal **(G)** views of a late divider. BC, buccal cirrus; DKA, dorsal kinety anlagen; EM, endoral membrane; FC, frontal cirrus; FM, frontal adoral membranelles; I–VI, frontal-ventral transverse anlagen; FVR, frontoventral row; LMR, left marginal row; Ma, macronuclear nodules; Mi, micronuclei; MP, midventral pair; MR, midventral row; PBC, parabuccal cirrus; PTC, pretransverse cirri; RB, replication band; RMR, right marginal row; TC, transverse cirri; VM, ventral adoral membranelles. Scale bars 30 μm.

**FIGURE 2:**
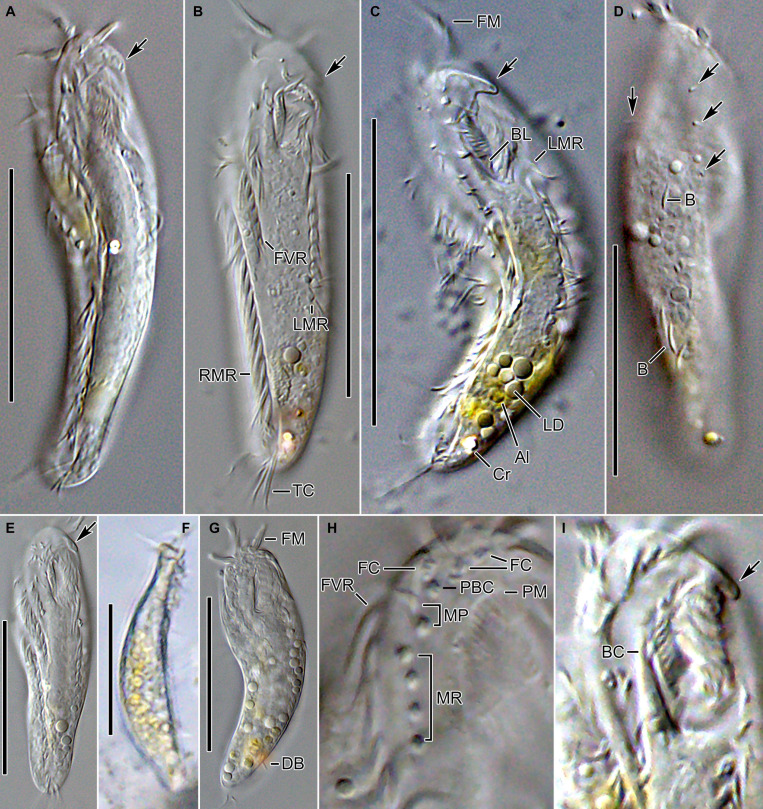
*Tunicothrix halophila* n. sp. *in vivo*. **(A–C,E)** Ventral views showing morphology. Arrows denote beak-like protrusion. **(D)** Dorsal view showing dorsal bristles (arrows) and arc-shaped cortical structures. **(F)** Lateral view. **(G)** Ordinary size of dorsal bristles. **(H)** Cirri on right frontoventral area. **(I)** Oral apparatus with beak-like protrusion (arrow). Al, algae; B, arc-shaped structures; BC, buccal cirrus; BL, left wall of buccal lip; Cr, cytoplasmic crystals; DB, dorsal bristles; FC, frontal cirri; FM, frontal adoral membranelles; FVR, frontoventral row; LD, lipid droplets; LMR, left marginal row; MP, midventral pair; MR, midventral row; PBC, parabuccal cirrus; PM, paroral membrane; RMR, right marginal row; TC, transverse cirri. Scale bars 30 μm.

**FIGURE 3:**
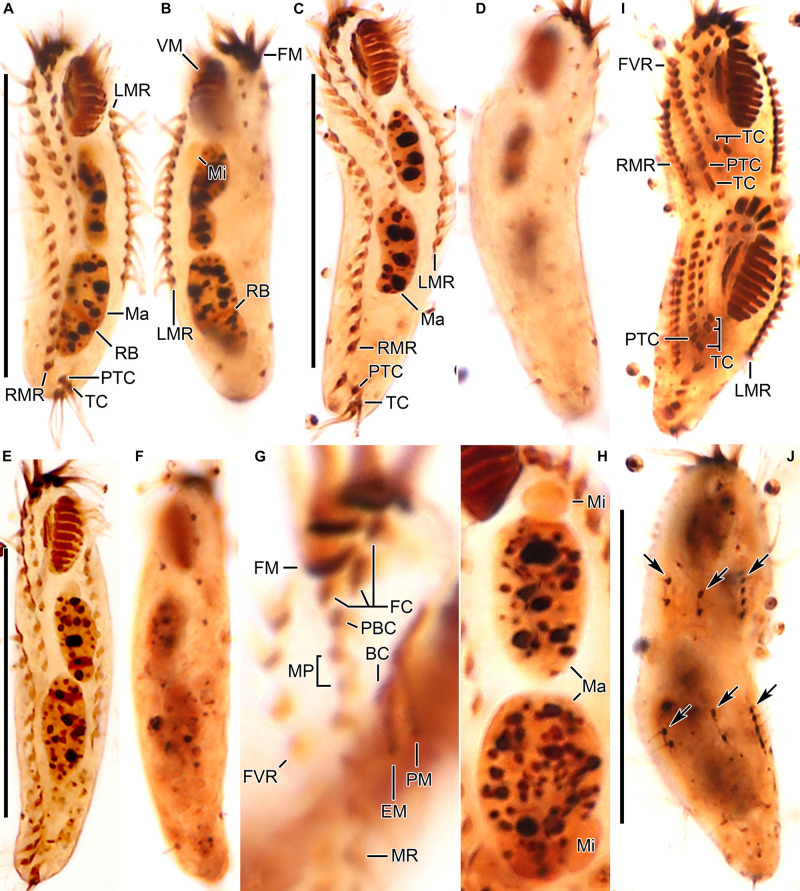
*Tunicothrix halophila* n. sp. after protargol impregnation. **(A,B)** Ventral **(A)** and dorsal **(B)** views of the holotype specimen. **(C–F)** Ventral **(C,E)** and dorsal **(D,F)** views. **(G)** Ventral view showing cirri on right frontoventral area. **(H)** Nuclear apparatus. **(I,J)** Ventral **(I)**, and dorsal **(J)** view of a late divider. Arrows denote dorsal kinety anlagen. BC, buccal cirrus; DB, dorsal bristles; EM, endoral membrane; FC, frontal cirri; FM, frontal adoral membranelles; FVR, frontoventral row; LMR, left marginal row; Ma, macronuclear nodules; Mi, micronuclei; MP, midventral pair; MR, midventral row; PBC, parabuccal cirrus; PM, paroral membrane; PTC, pretransverse cirri; RB, replication band; RMR, right marginal row; TC, transverse cirri; VM, ventral adoral membranelles. Scale bars 30 μm.

**FIGURE 4:**
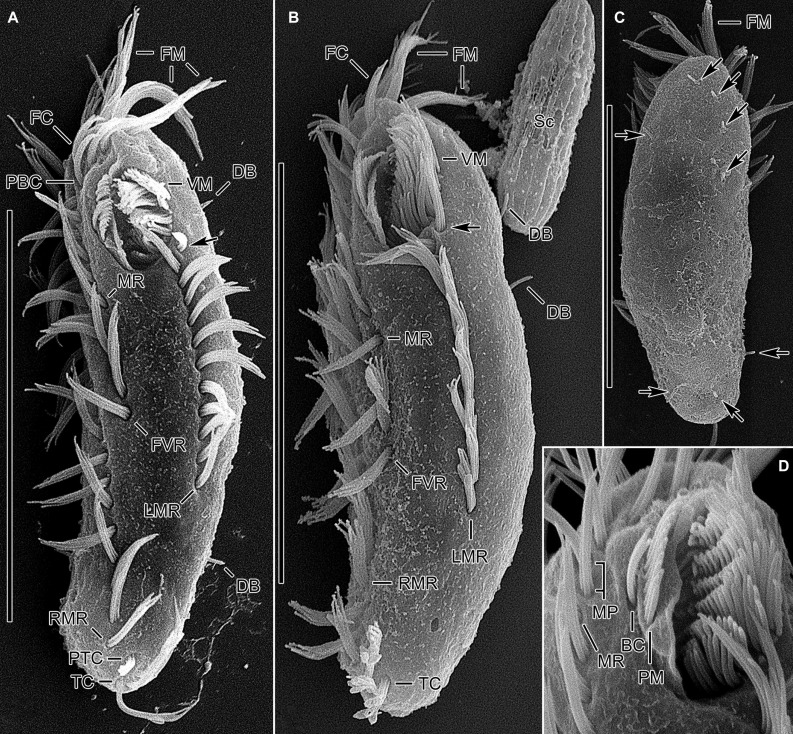
*Tunicothrix halophila* n. sp. in the scanning electron microscope. **(A,B)** Ventral and lateral view showing typical morphology. Arrows denote anteriormost left marginal cirrus curved leftward. **(C)** Dorsal view showing highly reduced dorsal bristles (arrows). **(D)** Oral apparatus. BC, buccal cirrus; DB, dorsal bristles; FC, frontal cirri; FM, frontal adoral membranelles; FVR, frontoventral row; LMR, left marginal row; MP, midventral pair; MR, midventral row; PBC, parabuccal cirrus; PM, paroral membrane; PTC, pretransverse cirri; RMR, right marginal row; Sc, unidentified scuticociliate; TC, transverse cirri; VM, ventral adoral membranelles. Scale bars 30 μm.

**FIGURE 5:**
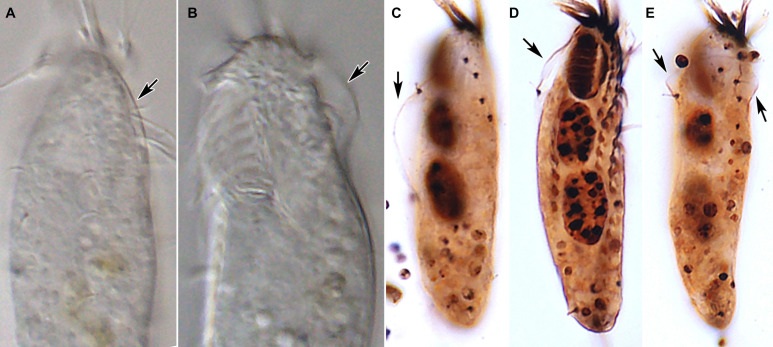
*Tunicothrix halophila* n. sp. *in vivo* **(A,B)** and after protargol impregnation **(C–E)**. Arrows denote the alveolar layer-like structure observed in only few cells.

Body size *in vivo* 45–70 × 12–18 μm (n = 12), on average 36.7 × 10.9 μm after protargol impregnation (Figures 1A,B, 2A,B, 3A–F, 4A–C). Body outline elliptical to slightly slender, posterior end narrower than anterior, dorsoventrally flattened (Figure 2F); body flexible but not contractile; cortex smooth except of ventral and ventrolateral grooves along frontoventral and marginal rows; beak-like protrusion between frontal and ventral adoral membranelles (Figures 2A–E,I); alveolar layer-like structure indistinct *in vivo*, not observed at magnifications of 50–400× (n = 50 cells), observed in some stained cells (3 out of 19 cells examined; Figure 5). Cells grayish to slightly yellowish at low magnification because of ingested algae (Figure 2C). Nuclear apparatus composed of two globular to ellipsoidal macronuclear nodules and one or two micronuclei slightly left of body midline (Figures 1A,D,E, 3A,C,E,H). Typical cortical granules lacking, but colorless arc-shaped structures underneath pellicle, 3.0–4.0 μm long *in vivo*, not observed in protargol-impregnated specimens (Figure 2D).

Cirri, except for buccal cirrus, 6–10 μm long *in vivo* and composed of four or six cilia (e.g., 2 × 2 or 2 × 3 arrangement) in SEM preparations, of them transverse cirri longest; buccal cirrus composed of three or four cilia in a row, 2–3 μm long in SEM preparations (Figures 2A–C,E, 4). Three frontal cirri, about 6–7 μm long; right frontal cirrus (III/1) below level of distal end of adoral zone of membranelles. Invariably one buccal cirrus right next to endoral membrane *in vivo* and 1 parabuccal (III/2) cirrus posterior to right frontal cirrus (Figures 1B,C, 2H,I, 3A,C,E,G, 4D). Midventral complex composed of one cirral pair and one cirral row; midventral pair terminates at level of buccal cirrus; midventral row composed of 3–5 cirri, extends to about 30% of body length. Frontoventral row (= right marginal row 1 in *Tunicothrix rostrata*) composed of 9–12 cirri, commences near level of parabuccal cirrus and terminates near mid-body. One pretransverse cirrus anterior and similar in size to transverse cirri; three transverse cirri arranged in J-shaped pattern. One left and one right marginal row; right row composed of 10–13 cirri, distinctly shortened anteriorly; left row, composed of 11–14 cirri, distinctly shortened posteriorly, anteriormost cirrus always slightly left to level of second cirrus.

Dorsal kineties very likely composed of three rows; sparsely ciliated (e.g., one or two bristles in row 1, one or two bristles in row 2, four to seven bristles in row 3; however, it should be noted that the “sparse” arrangement of bristles hampers accurate counting of the number of dorsal kineties); usually one bristle at anterior and/or posterior end of dorsal kineties 1 and 2; in dorsal kinety 3, usually four bristles anterior to mid-body and invariably one bristle at posterior body end; bristles about 2 μm long *in vivo*; caudal cirri lacking (Figures 1D,E,G, 2G, 3B,D,F,G, 4C).

Bipartite adoral zone (e.g., frontal and ventral membranelles) extends to about 25% of body length; DE value 0.29 on average; frontal and ventral zone invariably composed of 4 and 9 membranelles, respectively; cilia from frontal membranelles 7–12 μm long *in vivo* while those from ventral membranelles distinctly shorter (Figures 1B,C,E, 2A,C,E,G,I, 4A–C). Largest ventral membranelle composed of four ciliary rows and 3.3 μm wide in protargol preparations. Buccal cavity narrow and shallow. Undulating membranes slightly curved leftward and optically crossing at anterior region of endoral membrane; paroral membrane composed of one or two (slightly zigzaging) combined rows of basal bodies, cilia 2–3 μm long. Buccal lip flat-type; left wall of buccal lip distinct and 2–3 μm wide. Cytopharynx obliquely extending to right body margin in protargol preparations.

#### Late Divider

We found a late divider from protargol preparations (Figures 1F,G, 3I,J). The specimen shows six frontal-ventral-transverse cirral anlagen, of which the posteriormost cirri of anlagen IV–VI become pretransverse and transverse cirri. The midventral complex originates from anlagen IV (for midventral pair) and V (for midventral row). Three dorsal kinety anlagen for each daughter cell were developed. Considering the interphasic specimens (Table 1), new cilia very likely replace all parental ones; however, we cannot confirm the replacement of parental membranelles.

**TABLE 1:** Morphometric data of *Tunicothrix halophila* n. sp.

Characteristic^a^	Mean	*M*	*SD*	SE	CV	Min	Max	n
Body, length	36.7	36.5	4.0	0.9	10.9	30.2	47.7	21
Body, width	10.9	10.7	0.8	0.2	7.7	9.6	12.9	21
Body, length:width ratio	3.4	3.3	0.4	0.1	12.3	2.8	4.5	21
Adoral zone of membranelles, length	9.5	9.5	0.8	0.2	8.3	8.2	11.7	21
Body length:adoral zone of membranelles length, ratio	3.9	3.9	0.3	0.1	6.9	3.2	4.3	21
DE value	0.29	0.28	0.04	0.01	14.4	0.20	0.36	21
Frontal adoral membranelles, number	4.0	4.0	–	–	–	4.0	4.0	21
Ventral adoral membranelles, number	9.0	9.0	–	–	–	9.0	9.0	21
Adoral membranelles, width of largest base	3.3	3.4	0.2	0.0	5.6	2.8	3.6	21
Macronucleus nodules, number	2.0	2.0	–	–	–	2.0	2.0	21
Macronuclear nodule (anteriormost), length	7.7	7.7	1.4	0.3	17.5	6.2	10.8	21
Macronuclear nodule (anteriormost), width	4.4	4.4	0.7	0.2	16.9	3.0	5.9	21
Micronuclei, number	1.4	1.0	0.5	0.1	36.1	1.0	2.0	18
Micronucleus (random), length	2.7	2.6	0.3	0.1	12.4	2.2	3.2	18
Micronucleus (random), width	1.7	1.7	0.2	0.1	13.4	1.4	2.3	18
Frontal cirri, number	3.0	3.0	–	–	–	3.0	3.0	21
Buccal cirrus, number	1.0	1.0	–	–	–	1.0	1.0	21
Parabuccal cirrus, number	1.0	1.0	–	–	–	1.0	1.0	21
Midventral pair, number	1.0	1.0	–	–	–	1.0	1.0	21
Midventral row, number of cirri	4.1	4.0	0.4	0.1	9.7	3.0	5.0	20
Frontoventral row, number of cirri	10.4	10.0	0.7	0.1	6.4	9.0	12.0	22
Pretransverse cirrus, number	1.0	1.0	–	–	–	1.0	1.0	21
Transverse cirri, number	3.0	3.0	–	–	–	3.0	3.0	18
Left marginal row, number of cirri	10.7	11.0	0.7	0.2	6.7	10.0	13.0	21
Right marginal row, number of cirri	12.1	12.0	0.6	0.1	5.3	11.0	14.0	20
Dorsal kineties, number	3.0	3.0	–	–	–	3.0	3.0	20
Dorsal kinety 1, number of bristles	1.8	2.0	0.4	0.1	22.8	1.0	2.0	20
Dorsal kinety 2, number of bristles	1.6	2.0	0.5	0.1	32.9	1.0	2.0	20
Dorsal kinety 3, number of bristles	5.0	5.0	0.9	0.2	18.4	4.0	7.0	20
Dorsal kineties, a total number of bristles	8.4	8.0	0.8	0.2	9.7	7.0	10.0	20

**^*a*^Data based on protargol-impregnated specimens; all measurements in μm. CV, coefficient of variation (%); M, median; Max, maximum; Mean, arithmetic mean; Min, minimum; n, number of specimens examined; SD, standard deviation; SE, standard error of arithmetic mean.**

#### 18S rDNA Phylogeny

**FIGURE 6:**
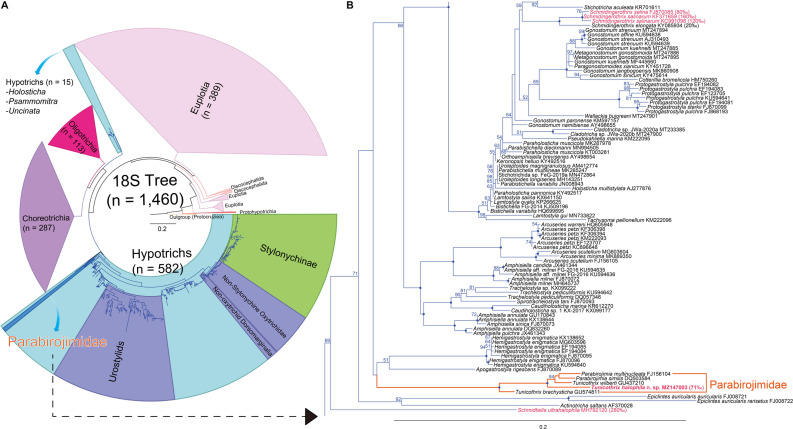
Maximum likelihood (ML) phylogenetic tree inferred from the 18S rRNA gene sequences **(A,B)**. **(A)** Complete tree comprising 1,460 sequences. **(B)** The clades containing the family Parabirojimidae and other halophile hypotrichs. Bootstrap values are shown near nodes, values ≤ 50% were not shown, dots at nodes represent 100 bootstrap value. Newly obtained sequence is in bold. Species from hypersaline habitats are in pink color. The scale bar represents 20 nucleotide substitutions per 100 nt.

The SSU rDNA sequence of *Tunicothrix halophila* n. sp. is 1,462 base pairs long and has a GC content of 46.7% (MZ147003). The pairwise similarities among the two congeners range from 96.66% (*T. brachysticha* GU574811) to 98.15% (*T. wilberti* GU437210). The family Parabirojimidae is monophyletic, and the sequence of *T. halophila* n. sp. shows a sister relationship to the clade composed of *T. wilberti* and *Parabirojimia* with full supporting value (Figure 6). However, the genus *Tunicothrix* is non-monophyletic because *Parabirojimia* sequences are nested within it. In addition, the clade of *P. multinucleata* and *P. similis* shows low supporting value (43) even though they cluster together. In other trees (data not shown) with small data sets, the genus *Parabirojimia* is paraphyletic because the clade composed of *T. halophila* n. sp. and *T. wilberti* are nested within it.

## Discussion

### Synonyms of “Right Marginal Row 1”

Many hypotrich ciliates (such as the gonostomatids, oxytrichids, and urostylids) are characterized by the presence of a cirral row which comprises few to many cirri on the right side of the buccal field and commences left and usually slightly ahead of the anterior end of the right marginal row. The terminology of this row depends on the number of cirri, its length, and its origin.

In the family Parabirojimidae, this row of cirri is long (usually as long as the right marginal row) and called “right ventral row” in *T.* wilberti (Lin and Song, 2004) and “right marginal row 1” in the other three species of *Tunicothrix* and *Parabirojimia* species (Hu et al., 2002; Xu et al., 2006; Chen et al., 2010; Dai and Xu, 2011). A similar row was called “frontoventral cirral row” in *Schmidingerothrix* (Lu et al., 2018). This row is represented by a few to many cirri in the gonostomatids and the core urostylids, while there are only two cirri in the oxytrichids called “frontoterminal cirri” (Berger, 1999, 2006, 2011; Foissner, 2016). On the other hand, the “amphisiellid median cirral row” definitely differs from that row because it is a mixed row composed of more than one true row. This organization is recognizable only during the ontogenetic process (Eigner and Foissner, 1994; Berger, 2008), and in his monograph, Berger (2008) used the neutral term “frontoventral row.”

Considering the ontogenesis, it seems that the origin of the hypotrich’s inner row (right marginal row 1) does not depend on its length. For instance, the long row of some hypotrichs originates intrakinetally (e.g., parabirojimids, schmidingerotrichids, and some gonostomatids) (Hu et al., 2002; Lin and Song, 2004; Wang et al., 2011, 2017; Foissner et al., 2014; Lu et al., 2018; present study) while in some other gonostomatids, it originates *de novo* or from oral primordium (e.g., *Gonostomum strenuum* (Song, 1990). However, this row could be considered a second (inner) marginal row if it originates intrakinetally and does not produce transverse cirri at the posterior end as in *Cladotricha*, *Parabistichella*, *Paragonostomoides*, and *Schmidingerothrix* (Berger, 2011; Foissner et al., 2014; Wang et al., 2017; Lu et al., 2018; Dong et al., 2020). On the other hand, it is considered a frontal-ventral-transverse row if it originates *de novo* (e.g., *Eschaneustyla*), from oral primordium (e.g., *Gonostomum strenuum*) or produces transverse cirrus despite its origin as in *Metagonostomum* and the parabirojimids (Hemberger, 1982, 1985; Song, 1990; Berger, 1999, 2006, 2011; Deng et al., 2018). Thus, we conclusively recommend using the neutral term “frontoventral row” for such row in the genus *Tunicothrix*.

### Comparison of *T. halophila* n. sp. With Closely Related Species

The genus *Tunicothrix* consists of four species as follows: *T. brachysticha* Dai and Xu, 2011; *T. multinucleata* Dai and Xu, 2011; *T. rostrata* Xu et al., 2006 (type species); and *T. wilberti* (Lin and Song, 2004) Xu et al., 2006. Considering the body size, the alveolar layer, and the numbers of cirri and membranelles, *T. halophila* n. sp. can be easily distinguished from these congeners by having the smallest body size and the least numbers of cirri and membranelles (Lin and Song, 2004; Xu et al., 2006; Dai and Xu, 2011).

Of the four congeners, *T. brachysticha* resembles *T. halophila* n. sp. However, as mentioned above, *T. brachysticha* differs from the new species by the body length (55.0–77.0 μm vs. 30.2–47.7 μm after protargol impregnation), the alveolar layer (distinct vs. absent/indistinct), and the numbers of frontal (6–9 vs. 4) and ventral (14–17 vs. 9) adoral membranelles, cirri in midventral row (8–13 vs. 3–5), left marginal cirri (17–30 vs. 10–13), right marginal cirri (16–22 vs. 11–14), and cirri in frontoventral row (16–22 vs. 9–12). In addition, they differ in the number (ordinary, i.e., equidistantly arranged in bipolar rows vs. reduced in *T. halophila* n. sp.) of dorsal bristles (Dai and Xu, 2011).

### Establishing a New Genus for *T. halophila* n. sp.

*Tunicothrix halophila* n. sp. differs from all other congeners by the absence/indistinctness (vs. presence) of the alveolar layer, which is a character state for the diagnosis/etymology of *Tunicothrix* (Xu et al., 2006). In addition, the highly reduced number of dorsal bristles (vs. ordinary in the type species *T. rostrata*) and the hypersaline habitat might be enough to establish a new genus. To justify the establishment of a new genus, however, further studies are needed on the following issues.

First, the genus *Tunicothrix* is non-monophyletic in the 18S rRNA gene tree (Zhang et al., 2018; Moon et al., 2020; the present study), i.e., *T. brachysticha* shows a basal position to the clade containing *Parabirojimia* and other *Tunicothrix* species. Second, the gene sequence of *T. brachysticha* (GU574811) was not obtained from the type population (Dai and Xu, 2011) that results in emphasizing a redescription of this species in terms of the morphology and DNA sequence simultaneously obtained from the same population. Third, we do not know the arrangement of dorsal bristles for *T. brachysticha* and *T. multinucleata* (Dai and Xu, 2011). All congeners including *T. halophila* n. sp. have three dorsal kineties, but Dai and Xu (2011) mentioned “dorsal kineties with sparse cilia” for the type population of *T. multinucleata*. Fourth, a detailed morphology of a Chinese population of *T. wilberti* (Huang et al., 2010; GenBank Acc. No. GU437210), which is necessary to clarify the non-monophyly of *Tunicothrix* in the gene tree (Figure 6), is unavailable. In addition, one (Wang et al., 2011) of the three populations of *T. wilberti* described from China (Lin and Song, 2004; Huang et al., 2010; Wang et al., 2011) differs from the type population by the widely (vs. ordinarily) spaced cilia (see Figure 4E in Wang et al., 2011). Further, the micrographs Figure 5 of Huang et al. (2010) and Figure 4F of Wang et al. (2011) likely belong to the same specimen. Thus, we cannot exclude that the populations of Huang et al. (2010) and Wang et al. (2011) are misidentifications.

In terms of the hypersaline habitat, *T. halophila* occurred at 71‰ while the other congeners are as follows: 31‰ for the type species/population *T. rostrata* (Xu et al., 2006); 18.5‰ for the type population of *T. wilberti* (Lin and Song, 2004), 20‰ for *T. wilberti* sensu Wang et al. (2011), salinity unavailable for *T. wilberti* sensu Huang et al. (2010); 29‰ for the type population of *T. brachysticha* (Dai and Xu, 2011); and 29‰ for the type population of *T. multinucleata* (Dai and Xu, 2011). The hypersaline habitat is very likely related with the reduction of dorsal bristles as in the genera *Apourosomoida* Foissner et al., 2002, *Erniella* Foissner, 1987, *Etoschothrix* Foissner et al., 2002, and *Schmidtiella* Li et al., 2019, or even the total loss of dorsal bristles as in *Schmidingerothrix* Foissner, 2012.

In conclusion, the reduction of dorsal ciliature in *T. halophila* n. sp. represents a diagnostic character state enough to establish a new genus. However, we refrain from establishing a new genus until the dorsal kinety pattern of other *Tunicothrix* species and populations is documented.

### Secondarily Oligomerized Dorsal Bristles

According to Berger (2008, 2011), the last common ancestor of the hypotrichs very likely had 18 frontal-ventral-transverse cirri originated from six cirral anlagen and three bipolar dorsal kineties each bearing a caudal cirrus. From our gene tree (Figure 6) and a previous report (Li et al., 2019), *Schmidtiella ultrahalophila* showed a basal position to most hypotrichs, and it has six cirral anlagen and one dorsal kinety. In addition, other basal taxa such as *Epiclintes* and *Actinotricha saltans* have more cirral anlagen and more dorsal kineties, respectively (Song et al., 1991; Hu et al., 2009). It is rather difficult to infer the last common ancestor of hypotrichs from the 18S rRNA gene because the supporting values of their clades are low, and they are non-monophyletic (see *Holosticha* in Figure 6A). Very likely, this is caused by undersampling, for instance, based on the recent redefinition on the Hypotrichia (Da Silva Paiva, 2020), the genus *Holosticha* forms a clade within hypotrichs, but with the increase of taxon sampling, it showed a sister relationship to the clade composed of Oligotrichia and Choreotrichia (Gao et al., 2016; Sheng et al., 2018).

Considering the reduced number of dorsal bristles, we can find some hypersaline hypotrichs at basal positions of the 18S rRNA gene tree (Foissner, 2012; Foissner et al., 2014; Lu et al., 2018; Li et al., 2019). These hypotrichs have very few or no dorsal bristles at all. According to Foissner (2012) and Remmert (1992), the reduction of the dorsal ciliature is very likely a modification caused by the hypersaline habitat. As shown in Figure 6, these hypersaline hypotrichs are definitely non-monophyletic, supporting the secondary oligomerization hypothesis. However, the interior branches on the basal taxa of the gene tree show lower supporting values even with the huge increase of taxon sampling, so further studies are necessary for basal hypotrichs or those living in hypersaline habitats to clarify their evolutionary relationship/history.

## Data Availability Statement

The datasets presented in this study can be found in online repositories. The names of the repository/repositories and accession number(s) can be found below: ZooBank.org; urn:lsid:zoobank.org:pub:7277C82F-18FF-483A-9747-BF22265B 0518; urn:lsid:zoobank.org:act:D64923EA-0756-4347-9051-D1B1ADAF53C8

## Author Contributions

AO carried out all laboratory work (preparations, analyses, micrographs, etc.). JHM collected the water sample and maintained the raw culture. AO, SWN, and J-HJ wrote the manuscript. All authors revised the manuscript.

## Conflict of Interest

The authors declare that the research was conducted in the absence of any commercial or financial relationships that could be construed as a potential conflict of interest.

## Footnotes

1 http://tree.bio.ed.ac.uk/software/figtree
